# Topologically Heterogeneous Beta Cell Adaptation in Response to High-Fat Diet in Mice

**DOI:** 10.1371/journal.pone.0056922

**Published:** 2013-02-18

**Authors:** Johanne H. Ellenbroek, Hendrica A. Töns, Natascha de Graaf, Cindy J. Loomans, Marten A. Engelse, Hans Vrolijk, Peter J. Voshol, Ton J. Rabelink, Françoise Carlotti, Eelco J. de Koning

**Affiliations:** 1 Department of Nephrology, Leiden University Medical Center, Leiden, The Netherlands; 2 Hubrecht Institute, Utrecht, The Netherlands; 3 Department of Molecular Cell Biology, Leiden University Medical Center, Leiden, The Netherlands; 4 Institute of Metabolic Science, University of Cambridge, Cambridge, United Kingdom; University of Bremen, Germany

## Abstract

**Aims:**

Beta cells adapt to an increased insulin demand by enhancing insulin secretion via increased beta cell function and/or increased beta cell number. While morphological and functional heterogeneity between individual islets exists, it is unknown whether regional differences in beta cell adaptation occur. Therefore we investigated beta cell adaptation throughout the pancreas in a model of high-fat diet (HFD)-induced insulin resistance in mice.

**Methods:**

C57BL/6J mice were fed a HFD to induce insulin resistance, or control diet for 6 weeks. The pancreas was divided in a duodenal (DR), gastric (GR) and splenic (SR) region and taken for either histology or islet isolation. The capacity of untreated islets from the three regions to adapt in an extrapancreatic location was assessed by transplantation under the kidney capsule of streptozotocin-treated mice.

**Results:**

SR islets showed 70% increased beta cell proliferation after HFD, whereas no significant increase was found in DR and GR islets. Furthermore, isolated SR islets showed twofold enhanced glucose-induced insulin secretion after HFD, as compared with DR and GR islets. In contrast, transplantation of islets isolated from the three regions to an extrapancreatic location in diabetic mice led to a similar decrease in hyperglycemia and no difference in beta cell proliferation.

**Conclusions:**

HFD-induced insulin resistance leads to topologically heterogeneous beta cell adaptation and is most prominent in the splenic region of the pancreas. This topological heterogeneity in beta cell adaptation appears to result from extrinsic factors present in the islet microenvironment.

## Introduction

The insulin producing pancreatic beta cells are essential to maintain blood glucose levels within a narrow range. When the demand for insulin is chronically increased by physiological or pathological changes, beta cells can adapt by enhancing insulin secretion via increased beta cell function and/or increased beta cell mass [1], [2]. Inadequate adaptation leads to the development of hyperglycemia and eventually diabetes mellitus [3], [4]. Therefore, insight into the mechanisms that control beta cell adaptation is important for developing therapies that can preserve or enhance beta cell mass.

The pancreas is a regionally heterogeneous organ. During embryonic development the pancreas originates from two epithelial buds. The ventral bud gives rise to the posterior part of the head and the uncinate process, and the dorsal bud forms the anterior part of the head, the body and the tail of the pancreas [5], [6]. Pancreatic islets derived from the ventral bud contain more cells producing pancreatic polypeptide (PP), whereas islets derived from the dorsal bud contain more alpha cells and secrete more insulin upon glucose stimulation [7], [8]. Furthermore, several histological studies in human pancreas describe a higher islet density in the tail compared to the body region of the pancreas [4], [9], [10].

While morphological and functional heterogeneity between individual islets exists, it is unknown whether there are regional differences in beta cell adaptation throughout the pancreas. Regional heterogeneity in cell proliferation rate is observed in regenerating liver lobules after partial hepatectomy [11]. In this study, we examine early events of beta cell adaptation in different regions of the pancreas using a model of high-fat diet induced insulin resistance in mice that is known to increase beta cell mass in the long term [12], [13].

## Research Design and Methods

### Animals

Male C57BL/6J mice, 8 weeks old (Charles River Laboratories, Wilmington, MA, USA) were fed a high-fat diet (HFD, 45 kcal% fat, D12451, Research Diets, New Brunswick, NJ, USA) or a normal diet (control, 10 kcal% fat, D12450B, Research Diets) for 6 weeks. Average food intake was determined per cage housing 3–4 mice weekly. For the 12-week diet study, 12 week old male C57BL/6J mice (Animal Facility Leiden University Medical Center), that were fed a high-fat or normal diet, were used. For islet transplantation experiments we used male C57BL/6J donor and recipient mice, 8–10 weeks old and fed regular chow. Animal experiments were approved by the ethical committee on animal care and experimentation of the Leiden University Medical Center (Permit Numbers: 09174, 07145, and 11146).

### Glucose and insulin tolerance test

An intra-peritoneal glucose tolerance test (GTT) was performed in overnight-fasted mice. Blood samples were drawn from the tail vein before injecting 2 g/kg glucose and after 15, 30, 60 and 120 minutes. An intra-peritoneal insulin tolerance test (ITT) was performed in animals that had been fasted for 6 hours. After measuring basal blood glucose concentration from the tail vein 0.75 U/kg insulin was injected followed by monitoring of the blood glucose concentrations after 15, 30 and 60 minutes. Blood glucose concentrations were measured using a glucose meter (Accu-Chek, Roche, Basel, Switzerland) and insulin concentrations were measured in 5 µl plasma samples by ELISA (Ultra Sensitive Mouse Insulin ELISA kit, Chrystal Chem, Downers Grove, IL, USA).

### Pancreas dissection and islet isolation

The pancreas was dissected, weighed and based on their spatial relation to adjacent organs divided into three parts: the duodenal, gastric and splenic region (Fig. S1) [14], [15]. The duodenal region was defined as the section of the pancreas attached to the duodenum, the gastric region as the part attached to the pylorus and stomach and the splenic region as the part attached to the spleen. For immunohistochemistry each pancreatic region was fixed in a random orientation in 4% paraformaldehyde and embedded in paraffin. For islet isolation the pancreas of 6–8 mice were pooled per region and digested using 3 mg/ml collagenase (Sigma-Aldrich, St Louis, CA, USA). Islets were manually picked and tested for insulin secretion or purified by gradient separation (1.077 g/ml ficoll, hospital pharmacy, LUMC) and transplanted after overnight culture.

### RNA preparation and real-time PCR

Total RNA was extracted from isolated islets using RNeasy micro kit (Qiagen, Hilden, Germany) according to the manufacturer's protocol. Total RNA (400 ng) was reverse transcribed using M-MLV reverse transcriptase (Invitrogen). Quantitative PCR (qPCR) was performed on a CFX384 Real-Time PCR Detection System (Bio-Rad Laboratories, Hercules, CA, USA) using the SYBR Green PCR Master Mix (Applied Biosystems, Foster city, CA, USA). Fold induction was calculated using deltaCT method with mouse cyclophilin as housekeeping gene. Mouse primers used were: cyclophilin (forward) 5'-CAGACGCCACTGTCGCTTT-3' and (reverse) 5'-TGTCTTTGGAACTTTGTCTGCAA-3'; cyclin D1 (forward) 5'- TCCGCAAGCATGCACAGA-3' and (reverse) 5'-GGTGGGTTGGAAATGAACTTCA-3'; insulin 2 (forward) 5'-CTGGCCCTGCTCTTCCTCTGG-3' and (reverse) 5'CTGAAGGTCACCTGCTCCCGG-3'.

### Glucose-induced insulin secretion

Groups of 10 islets were incubated in a modified Krebs-Ringer Bicarbonate buffer (KRBH) containing 115 mM NaCl, 5 mM KCl, 24 mM NaHCO_3_, 2.2 mM CaCl_2_, 1 mM MgCl_2_, 20 mM HEPES, 2 g/l human serum albumin (Cealb, Sanquin, The Netherlands), pH 7.4. Islets were successively incubated for 1 hour in KRBH with 2 mM and 20 mM glucose at 37°C. Insulin concentration was determined in the supernatants by ELISA (Mercodia, Uppsala, Sweden). Insulin secretion was corrected for DNA content to correct for islet size differences. DNA content was determined by Quant-iT PicoGreen dsDNA kit (Invitrogen, Carlsbad, CA, USA).

### Islet transplantation

For islet transplantation experiments recipient mice were made diabetic by intra-peritoneal injection of 160 mg/kg streptozotocin (STZ, Sigma-Aldrich), freshly dissolved in citrate buffer (pH 4.5). Mice were considered diabetic when the blood glucose concentration was greater than 20 mmol/L. Blood glucose concentrations were determined in blood obtained from the tail vein by a glucose meter (Accu-Chek). Prior to the transplantation mice were given 0.1 mg/kg buprenorfin (Temgesic, Schering-Plough, Kenilworth, NJ) after which they were anaesthetized using isoflurane and kept warm on a heating pad. The left kidney was exposed by a small opening in the flank of the mouse. A small incision was made in the kidney capsule. Using a Hamilton syringe (Hamilton Company, Reno, CA) and polyethylene tubing (PE50, Becton Dickinson, Franklin Lakes, NJ) siliconized with Sigmacote (Sigma-Aldrich), 150 islets per mouse were transplanted under the kidney capsule. The peritoneum and the skin were sutured and the animals were allowed to recover under a warm lamp. Blood glucose concentrations were monitored every other day after transplantation via blood from the tail vein. The islet graft was removed 10 days post-transplantation, fixed by immersion in a 4% paraformaldehyde solution, embedded in paraffin blocks and sliced into 4 µm sections and mounted on slides.

### Beta cell mass morphometry and proliferation

For the identification of beta cells, sections were immunostained with guinea-pig anti-insulin IgG (Millipore, Billerica, MA, USA) or rabbit anti-insulin IgG (Santa Cruz Biotechnology, Santa Cruz, CA, USA) for 1 hour followed by HRP- or AP- conjugated secondary antibodies for 1 hour. Sections were developed with 3,3'-diaminobenzidine tetrahydrochloride (DAB) or liquid permanent red (LPR, Dako, Denmark) and counterstained with hematoxylin.

For determining the beta cell mass, 3-4 insulin-DAB stained sections (200 µm apart) per pancreatic region were digitally imaged (Panoramic MIDI, 3DHISTECH, Hungary). Beta cell area and pancreas area stained with hematoxylin were determined using an image analysis program (Stacks 2.1, LUMC), excluding large blood vessels, larger ducts, adipose tissue and lymph nodes. The area of clusters containing ≥4 beta cells was individually measured and used to determine the average beta cell cluster area per pancreatic region. Islet density was determined by dividing the number of beta cell clusters by the (regional) area that was analyzed. Beta cell mass was determined by the percentage of beta cell area to pancreas (regional) area multiplied by the pancreas (regional) weight.

Two techniques were used to identify proliferating beta cells. First, incorporation of 5-bromo-2′-deoxyuridine (BrdU, Sigma-Aldrich) in proliferating beta cells was established by administering 50 mg/kg BrdU subcutaneously twice daily during the final 7 days of the 6-week study period. The transplanted recipient mice received 1 mg/ml BrdU in the drinking water (refreshed every other day) during the final 7 days. Sections were double stained for insulin-LPR and BrdU (BrdU staining kit, Invitrogen). BrdU-positive beta cells were assessed as a proportion of all beta cells per pancreatic region or islet graft. Second, sections were double stained with goat anti-Ki67 IgG (Santa Cruz Biotechnology) and guinea-pig anti-insulin IgG (Millipore) overnight at 4°C after heat-induced antigen retrieval in 0.01 M citrate buffer (pH 6.0) followed by biotin-conjugated anti-goat IgG (Dako), streptavidine-Alexa 488 (Invitrogen) and TRITC-conjugated anti-guinea-pig (Jackson ImmunoResearch Laboratories, West Grove, PA, USA). Nuclei were stained with DAPI (Vector Laboratories, Burlingame, CA, USA). Apoptotic beta-cells were counted after being identified by immunostaining for insulin and by the terminal deoxynucleotidyl-transferase-mediated deoxyuridine 5-triphosphate nick end labeling (TUNEL) assay (Roche). The investigator was blind to the experimental conditions.

### Statistical analysis

Data are presented as means ± SEM. Statistical calculations were carried out using GraphPad Prism 5 (GraphPad Software, San Diego, CA, USA). The statistical significance of differences was determined by an unpaired Student's *t* test or two-way ANOVA, followed by Bonferroni's multiple comparisons test, as appropriate. *P*<0.05 was considered statistically significant.

## Results

### Metabolic characteristics of mice fed HFD for 6 weeks

**Figure 1:**
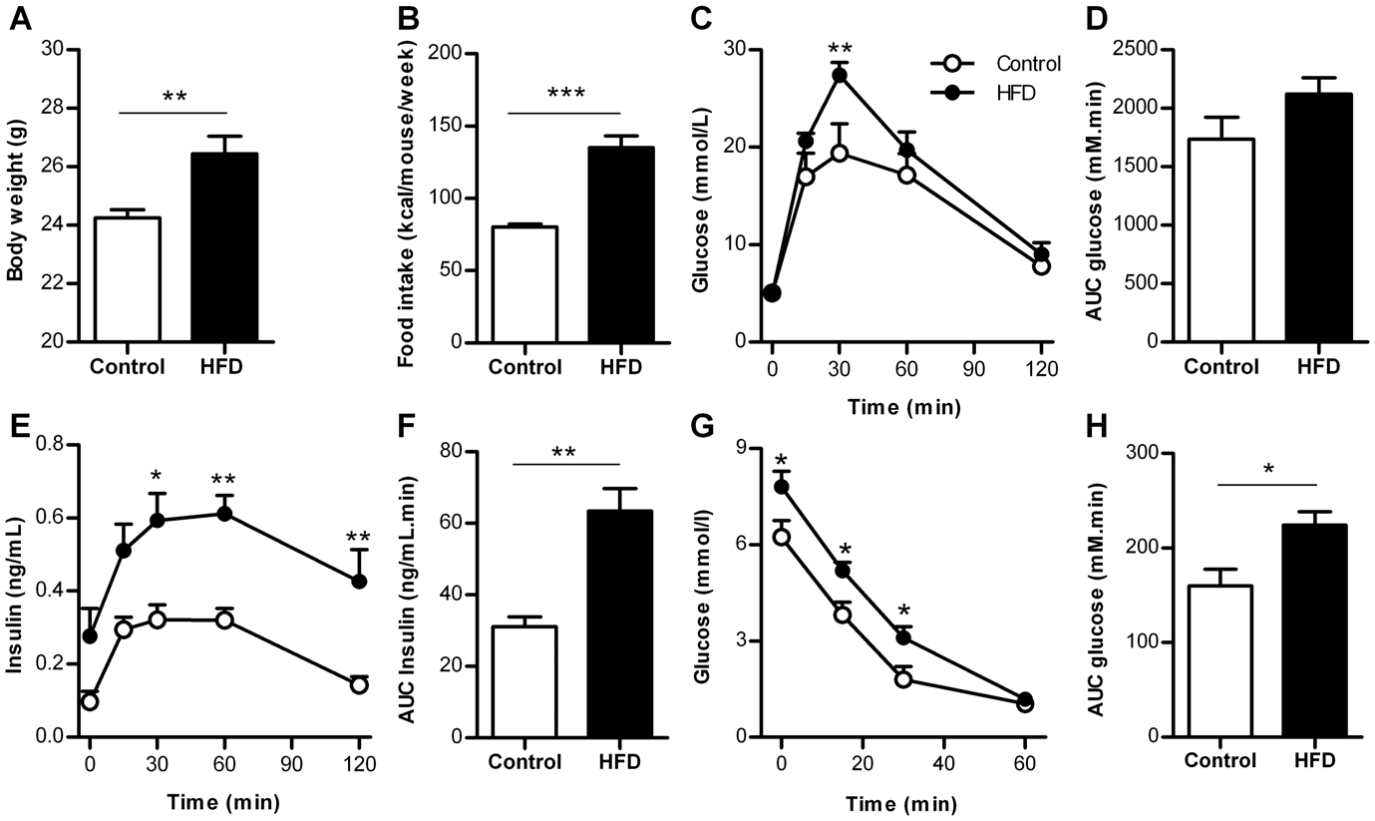
Metabolic characteristics of control mice and mice fed a high fat diet for 6 weeks. A. Body weight (n = 13–14 mice). B. Food intake (n = 4 cages). C. Blood glucose concentrations during GTT (n = 6 mice). D. AUC blood glucose concentrations during GTT (n = 6 mice). E. Insulin concentrations during GTT (n = 5–6 mice). F. AUC insulin concentrations during GTT (n = 5–6 mice). G. Blood glucose concentrations during ITT (n = 8 mice). H. AUC of glucose concentrations during ITT (n = 8 mice). HFD  =  high-fat diet, AUC  =  area under the curve. *p<0.05, **p<0.01, ***p<0.001.

Body weight and food intake were increased after 6 weeks HFD compared to control (Fig. 1A, B). After overnight fasting glucose concentrations were similar (5.1±0.2 mmol/L (HFD) vs 5.0±0.2 mmol/L (control), *p* = 0.72). Glucose tolerance was decreased in HFD mice compared to control (Fig. 1C, D), whereas insulin concentrations were increased twofold (Fig. 1E, F). Insulin tolerance was decreased by HFD (Fig. 1G, H). Therefore, HFD for 6 weeks is sufficient to induce insulin resistance leading to an increased demand for insulin from beta cells.

### Increased beta cell proliferation in the splenic region of the pancreas in response to HFD

**Figure 2:**
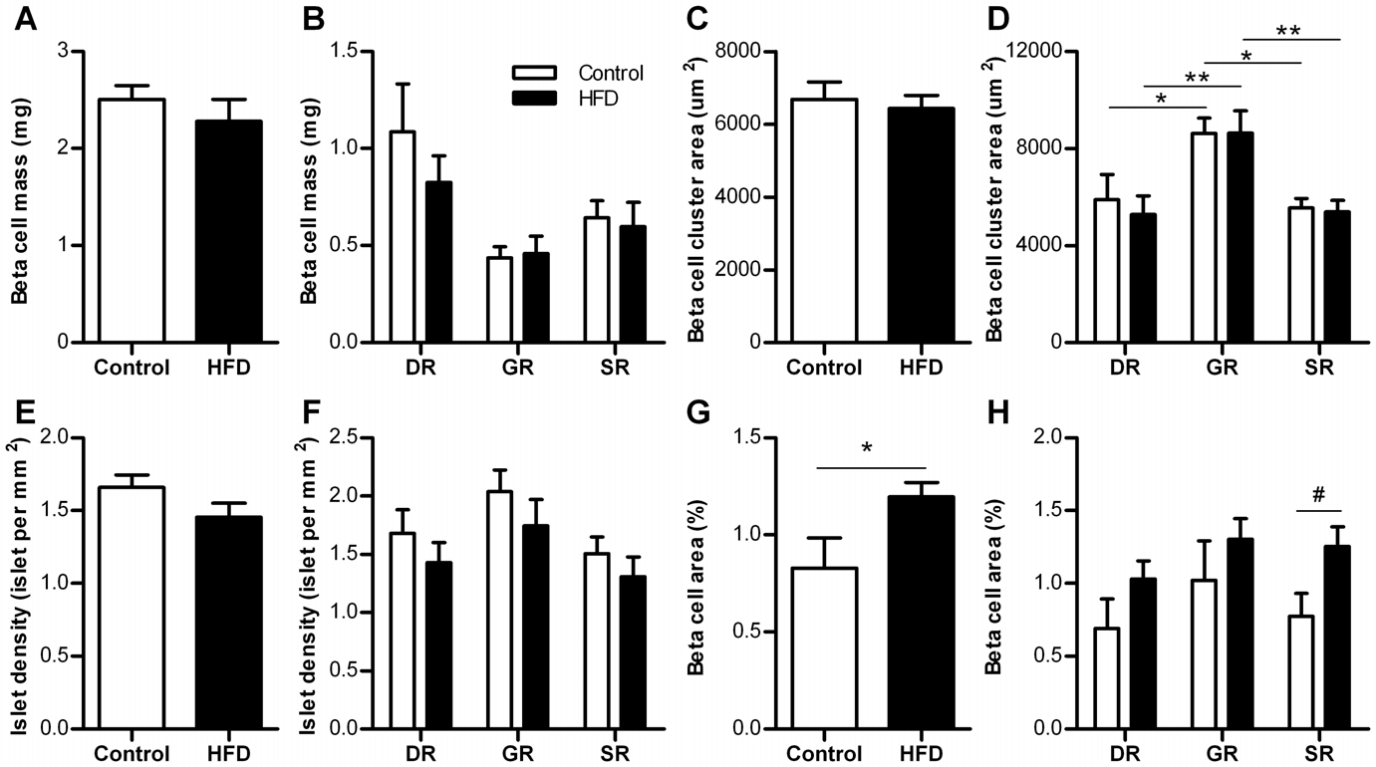
Beta cell mass morphometry in control and HFD mice. A. Beta cell mass in the entire pancreas after 6 weeks (n = 6 mice). B. Beta cell mass by pancreatic region after 6 weeks (n = 6 mice per region). C. Beta cell cluster area in the entire pancreas after 6 weeks (n = 6 mice). D. Beta cell cluster area by pancreatic region after 6 weeks (n = 6 mice per region). E. Islet density in the entire pancreas after 6 weeks (n = 6 mice). F. Islet density by pancreatic region after 6 weeks (n = 6 mice per region). G. Beta cell area in the entire pancreas after 12 weeks (n = 6 mice). H. Beta cell area by pancreatic region after 12 weeks (n = 6 mice per region). DR  =  duodenal region, GR  =  gastric region, SR  =  splenic region, HFD  =  high-fat diet. *p<0.05, ^#^ *p*<0.05 by unpaired Student's *t* test.

The effect of HFD on early beta cell adaptation in different regions was evaluated. The pancreas was divided in three regions: a duodenal, gastric and splenic region. The beta cell mass was determined by analyzing 29.9±1.6 mm^2^ pancreatic tissue per region per mouse. A difference in beta cell mass between HFD and control mice was found neither in the entire pancreas, nor in the separate regions (Fig. 2A, B). For determination of the average beta cell cluster area 46±2.5 clusters were included per region. The beta cell cluster area was significantly larger in islets from the GR, no differences were found between HFD and control mice (Fig. 2C, D). Islet density was homogeneous throughout the pancreas and similar after HFD for 6 weeks (Fig. 2E, F). After 12 weeks HFD we could confirm an increased beta-cell area, which was mostly augmented in the SR of the pancreas compared to control mice (Fig. 2G, H).

**Figure 3:**
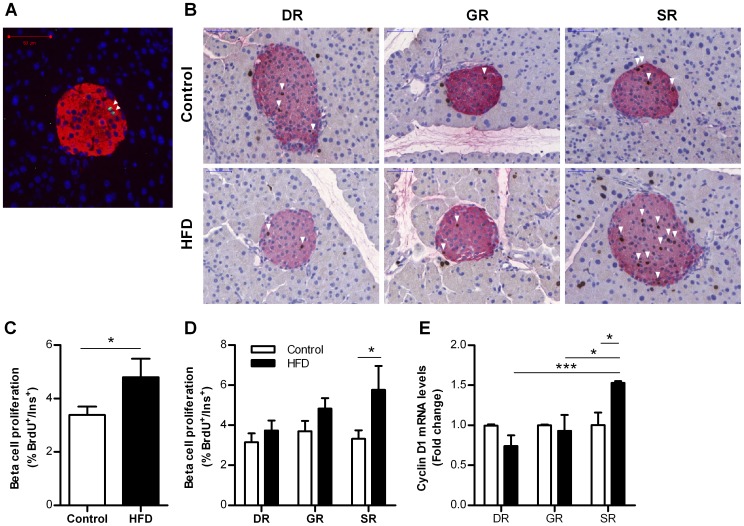
Beta cell proliferation in control and HFD mice after 6 weeks. A. Image of proliferating beta-cells (arrowheads), Ki67 (green), insulin (red) and DAPI (blue). Scale bar = 50 µm. B. Image of proliferating beta-cells (arrowheads), BrdU (brown) and insulin (red) per pancreatic region in control and HFD mice. Mice received BrdU during the final 7 days. Scale bar = 50 µm. C. Beta cell proliferation in the entire pancreas, BrdU labeling during the final 7 days (n = 6 mice). D. Beta cell proliferation by pancreatic region, BrdU labeling during the final 7 days (n = 6 mice per region). E. Cyclin D1 mRNA expression by pancreatic region, control = 1. DR  =  duodenal region, GR  =  gastric region, SR  =  splenic region, HFD  =  high-fat diet. *p<0.05, ***p<0.001

For determination of the number of proliferating beta cells Ki67+ beta cells were counted in 95±6 islets per mouse (Fig. 3A). The occurrence of Ki67+/insulin+ cells was very low (Ki67+/Insulin+ 0.12±0.03% (HFD) vs. 0.09±0.02% (control)). To increase the sensitivity for detecting proliferating beta cells, BrdU was administered for 7 days. We counted 885±48 beta cells per region per mouse. Beta cell proliferation was significantly increased in HFD mice compared to control mice (Fig. 3C). A positive correlation was found between the increase in body weight and the rate of beta cell proliferation in HFD mice (r^2^ = 0.70; *p*<0.05) (data not shown). HFD increased beta cell proliferation by 70% in the SR of the pancreas whereas no significant increase was found in the DR and GR (Fig. 3B, D). Also mRNA levels of Cyclin D1 were increased in islets from the splenic region of HFD mice (Fig. 3E). By counting on average 460±73 cells per region per mouse, the occurrence of apoptotic beta cells was very low and not different between the two groups (data not shown).

### Prominent increase in glucose-induced insulin release from isolated islets in the splenic region by HFD

**Figure 4:**
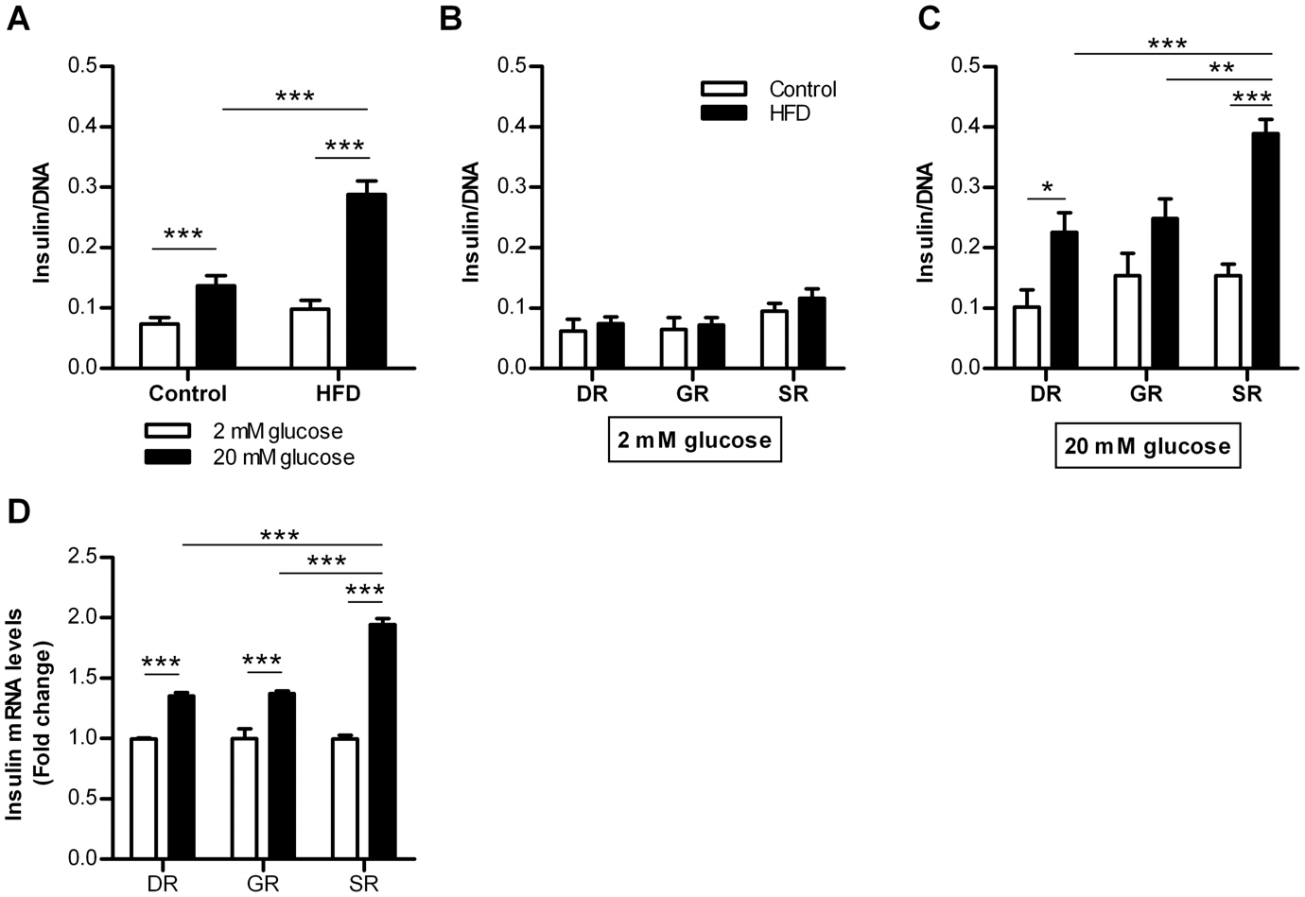
Glucose-induced insulin secretion from isolated islets of control and HFD mice. Insulin secretion was corrected for DNA content. A. Insulin secretion during 2 mM and 20 mM glucose stimulation from islets in the entire pancreas (n = 24). B. Insulin secretion from islets by pancreatic region during incubation in 2 mM glucose buffer (n = 8 per region) and C. 20 mM glucose buffer (n = 8 per region) for control and HFD mice. D. Insulin mRNA expression by pancreatic region, control = 1. DR  =  duodenal region, GR  =  gastric region, SR  =  splenic region, HFD  =  high-fat diet. *p<0.05, **p<0.01, ***p<0.001.

The functional adaptation of islets from HFD mice was assessed by measurement of glucose-induced insulin secretion. Stimulation of islets from HFD mice with 20 mM glucose led to a twofold increase in insulin secretion compared to control mice (Fig. 4A). When comparing the response of islets derived from the different pancreatic regions after HFD, insulin secretion was 56% and 72% higher in SR islets compared to GR and DR islets, respectively (Fig. 4B, C). Expression levels of Insulin mRNA showed a similar pattern (Fig. 4D).

### Similar islet function and beta cell proliferation after transplantation of islets from different regions to an extrapancreatic location

**Figure 5:**
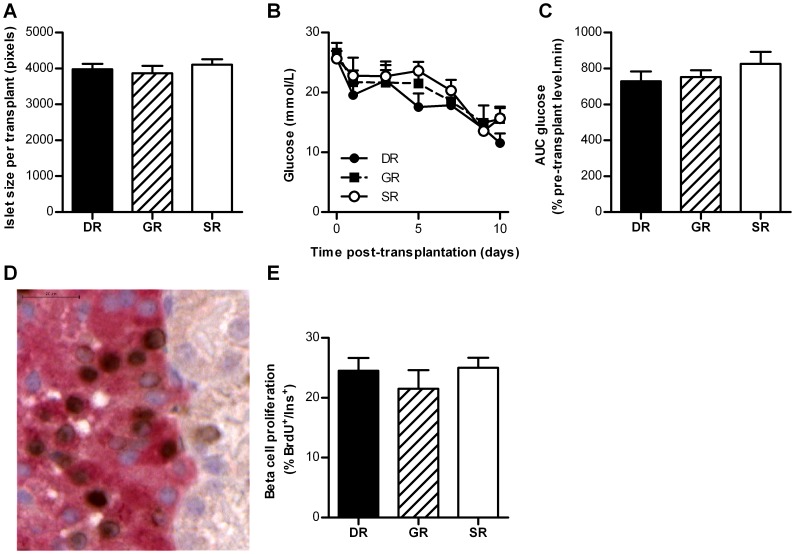
Beta cell adaptation in islets grafts from different pancreatic regions transplanted in syngeneic diabetic mice. A. Average islet size per transplant. B. Blood glucose concentrations of STZ-induced diabetic mice followed up to 10 days after transplantation (n = 6–8 mice per region) of DR, GR or SR islets. C. AUC blood glucose concentrations post-transplantation corrected for pre-transplantation glucose concentration (n = 6–8 mice per region). D. Image of proliferating beta-cells, positive for both BrdU (brown) and insulin (red) in islets transplanted under the kidney capsule of diabetic mice. Scale bar = 20 µm. E. Beta cell proliferation in the islet grafts 10 days after transplantation, BrdU labeling during the final 7 days (n = 6–7 mice per region). DR  =  duodenal region, GR  =  gastric region, SR  =  splenic region, AUC  =  area under the curve.

Next we assessed whether the observed proliferative and functional islet heterogeneity is due to differences in the islet microenvironment or due to intrinsic differences between islets from the three regions. Islets isolated from the three pancreatic regions were transplanted under the kidney capsule of syngeneic STZ-induced diabetic mice. All grafts contained 150 handpicked islets with an average size of that was similar for all transplants resulting in a similar graft size (Fig. 5A). Since hyperglycemia in these mice is required for detectable adaptation of grafted islets [16], [17], the increased demand for insulin in this model is expected to be a potent stimulus for beta cell adaptation in the islet graft. The islet graft size was sufficient to reduce blood glucose concentrations, but it was not sufficient to lead to normoglycemia in most mice thereby maintaining the stimulus for beta cells to adapt [17]. Normoglycemia (defined as blood glucose concentration <10 mmol/L) was reached with 3 out of 7 DR, 1 out of 6 GR and 2 out of 8 SR islet grafts. On average, all transplants led to a similar decrease in hyperglycemia (blood glucose concentrations 10 days post-transplantation 11.5±1.6 mmol/L (DR grafts), 15.5±2.1 mmol/L (GR grafts), 15.7±1.7 (SR grafts) mmol/L, *p* = 0.20; Fig. 5B, C). For determination of the number of proliferating beta cells in the grafts 1114±113 beta cells per islet graft were counted. Beta cell proliferation was similar for DR, GR or SR islet grafts (*p* = 0.53, Fig. 5D, E).

## Discussion

The main results of our study show that beta cell adaptation is topologically heterogeneous throughout the pancreas. Splenic islets are involved in the first line of response in beta cell adaptation. Although morphological and functional heterogeneity between individual islets have been described before, this is the first study showing regional differences in beta cell adaptation to an increased metabolic demand.

Beta cell adaptation in different pancreatic regions was studied in mice fed HFD for 6 weeks. We hypothesized that this time period would be long enough to induce metabolic changes and would allow us to investigate early islet adaptation. Six weeks HFD led to insulin resistance, with a higher demand for insulin to which beta cells started to adapt. Since beta cell mass was not significantly changed yet, but an increased rate of beta cell proliferation was already observed, this is an appropriate model for studying early events of beta cell adaptation.

The presence of increased beta cell proliferation and an augmented insulin secretory response in islets derived from the splenic region of the pancreas indicates that islets in this part of the pancreas constitute an early line of defense against an increased insulin demand. Our study was not designed to answer whether the relative contribution of beta cell adaptation in the different regions changes during prolonged high-fat feeding.

The observed proliferative and functional heterogeneity between islets from different regions in response to a HFD stimulus could be explained in two ways: either the islets from different pancreatic regions are intrinsically different or they receive distinct extrinsic signals from their microenvironment. This latter hypothesis was investigated by transplanting isolated islets to an extrapancreatic location in diabetic mice. After 10 days, islet grafts from the duodenal, gastric or splenic region led to a similar decrease in hyperglycemia and there was no difference in beta cell proliferation. Therefore we suggest that this newly identified topological heterogeneity of beta cell adaptation observed in HFD mice is most likely the result of distinct extrinsic signals present in the microenvironment of the islet.

The islet microenvironment is formed by a complex network of nerves and blood vessels that mediate neuronal, humoral and circulatory signals which are involved in beta cell adaptation. Islets are densely innervated by the autonomic nervous system [18] and it was reported that beta cell mass adaptation is regulated by neuronal signals from the liver [19]. A recent study identified a subpopulation (5%) of islets with greater blood perfusion and vascular density, which was associated with increased beta cell function and proliferation [20]. But it still remains unclear whether the increased vascular density is the cause or the consequence of the increased beta cell mass. Another possible factor is the strong paracrine dialogue between the islet microvasculature and beta cells [21]. However, here we show that early adaptation persists *in vitro* after isolation of the islets, since SR islets from HFD mice display an enhanced glucose-induced insulin secretion compared to DR and GR islets. Therefore, this indicates that heterogeneity in beta cell adaptation is not dependent on immediate innervation or vascular blood supply.

Furthermore, a strong structural and functional relationship between islets and acinar cells exists, which is referred to as the islet-acinar axis, in which insulin and somatostatin play an important role in regulating exocrine function [22]. It was shown that the amylase content of acinar tissue from the splenic region of rats is higher than in the duodenal region of the pancreas [23]. Whether the exocrine tissue surrounding islets can locally influence beta cell adaptation remains an open question.

Past studies have shown that islets from the dorsal pancreas secrete more insulin compared to islets from the ventral region [8], [24]. The gastric and splenic regions originate from the dorsal lobe. However our data also show heterogeneous adaptation within the dorsal pancreas (GR vs SR) indicating that embryonic origin of the different regions does not entirely explain the heterogeneity observed in this study.

It is likely that our findings of regional heterogeneity in beta cell adaptation can be extended to the human pancreas. In the pancreas of humans and non-human primates heterogeneity in islet density throughout the pancreas [4], [9], [10], and changes in islet mass associated with different metabolic conditions have been described [3], [25]–[29]. For patients undergoing distal pancreatectomy, this would imply loss of the most adaptive islets which may lead to a higher risk for postoperative diabetes. Furthermore, histological studies of beta cell adaptation in the human pancreas are often based on tissue samples from the splenic region only [3], [25], whereas this may not be representative for the entire organ.

Finally, the findings of this study imply that the islet microenvironment harbors factors that are involved in beta cell adaptation. Investigation of these regional differences may lead to the identification of factors that play a key role in beta cell regeneration.

## Supporting Information

Figure S1 **The spatial relation to adjacent organs was used to divide the pancreas into three parts: DR  =  duodenal region, GR  =  gastric region, SR  =  splenic region.**
(TIF)
