# Supplementary figures and images for: Topologically Heterogeneous Beta Cell Adaptation in Response to High-Fat Diet in Mice
Source: PLoS One. 2013 Feb 18;8(2):e56922. doi: 10.1371/journal.pone.0056922 (PMC3575501; doi:10.1371/journal.pone.0056922)

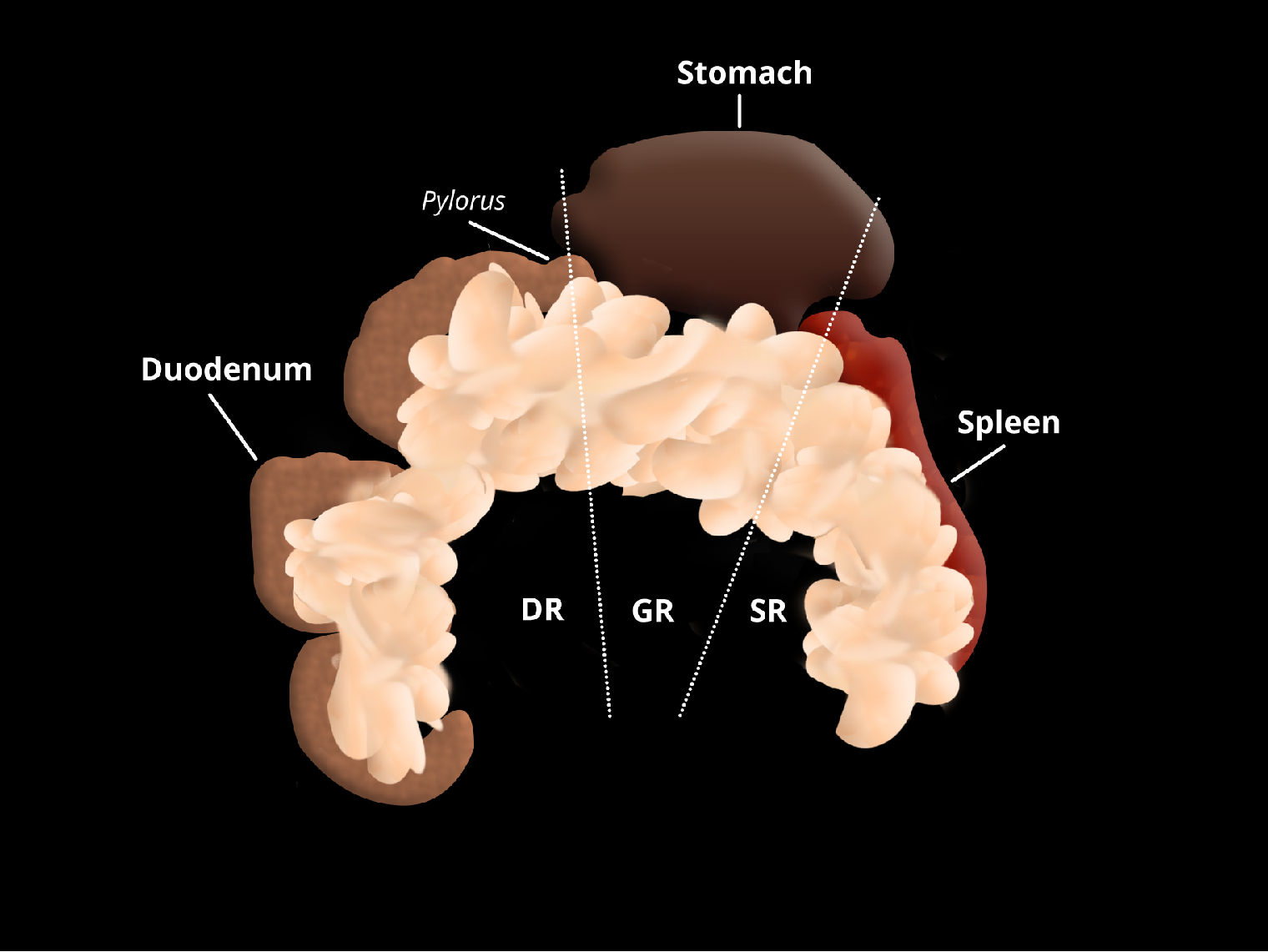

Supplement: Figure S1 — The spatial relation to adjacent organs was used to divide the pancreas into three parts: DR = duodenal region, GR = gastric region, SR = splenic region. (TIF) [file pone.0056922.s001.tif]
